# Dose Conversion Ratio Between Roxadustat and Daprodustat in Patients With Non-dialysis Chronic Kidney Disease

**DOI:** 10.7759/cureus.114463

**Published:** 2026-08-13

**Authors:** Keiji Hirai, Junki Morino, Katsunori Yanai, Yuko Mutsuyoshi, Taisuke Kitano, Haruhisa Miyazawa, Kiyonori Ito, Takahiro Masuda, Susumu Ookawara, Yoshiyuki Morishita

**Affiliations:** 1 Division of Nephrology, Department of Internal Medicine, Jichi Medical University, Tochigi, JPN; 2 Division of Nephrology, First Department of Integrated Medicine, Saitama Medical Center, Jichi Medical University, Saitama, JPN

**Keywords:** anemia, chronic kidney disease, daprodustat, hif-ph inhibitor, roxadustat

## Abstract

Objective: This study aimed to determine the dose conversion ratio between roxadustat and daprodustat in non-dialysis chronic kidney disease (CKD) patients by switching from roxadustat to daprodustat.

Methods: Changes in hemoglobin, serum ferritin, transferrin saturation, and the doses of roxadustat and daprodustat were retrospectively analyzed in 15 patients (mean hemoglobin concentration: 11.2 ± 0.9 g/dL; mean estimated glomerular filtration rate: 14.3 ± 9.4 mL/min/1.73 m^2^; CKD stage G3b: 1, stage G4: 4, stage G5: 10) during the 12 months before and after switching from roxadustat to daprodustat.

Results: Hemoglobin, serum ferritin concentrations, and transferrin saturation did not change after switching from roxadustat to daprodustat. The dose of roxadustat before switching was 51 ± 18 mg/administration, whereas the dose of daprodust at 12 months after switching was 5.2 ± 2.8 mg/administration. Accordingly, the dose conversion ratio between roxadustat and daprodustat was approximately 10:1 (51 ÷ 5.2 ≈ 10).

Conclusion: Our study results suggest that the dose conversion ratio between roxadustat and daprodustat is approximately 10:1 in non-dialysis CKD patients.

## Introduction

Anemia is one of the commonest complications in chronic kidney disease (CKD) patients, and it is reportedly associated with an accelerated decline in renal function, impaired quality of life, and higher risk of mortality [1-3]. Therefore, appropriate treatment for anemia is important to preserve renal function and quality of life and improve the life prognosis of CKD patients.

Hypoxia-inducible factor prolyl hydroxylase (HIF-PH) inhibitors are novel orally available agents that enhance endogenous erythropoietin production through the stabilization of HIF by inhibiting the activity of the HIF-PH domain [4]. Roxadustat has been reported to be superior to other HIF-PH inhibitors with respect to its effect on anemia in CKD patients [5]. Daprodustat has been shown to have less drug-drug interaction potential and can be administered with cation-containing agents [6]. Thus, these two HIF-PH inhibitors are considered potent therapeutic agents for the treatment of anemia in CKD. However, there is little data regarding the dose conversion ratio between roxadustat and daprodustat. Therefore, in this study, we determined the dose conversion ratio between roxadustat and daprodustat in non-dialysis CKD patients by switching from roxadustat to daprodustat.

## Materials and methods

Ethics approval and informed consent

This study was approved by the Ethics Committee of Saitama Medical Center, Jichi Medical University (RIN 22-004), and it complied with the basic principles of the Helsinki Declaration. Since this study was a retrospective study, informed consent was not required by the Ethics Committee. Instead, the opportunity to opt out of the study was provided to the participants.

Participants

We retrospectively analyzed the data of the patients who had been treated between 2020 and 2023. Inclusion criteria were: (i) age ≥ 20 years; (ii) estimated glomerular filtration rate (eGFR) < 60 mL/min/1.73 m^2^ (CKD stage G3-5 [7]); and (iii) a history of taking daprodustat for at least 12 months after taking roxadustat for at least 12 months. The exclusion criterion was renal replacement therapy, namely hemodialysis, peritoneal dialysis, and renal transplantation.

Study design

**Figure 1:**
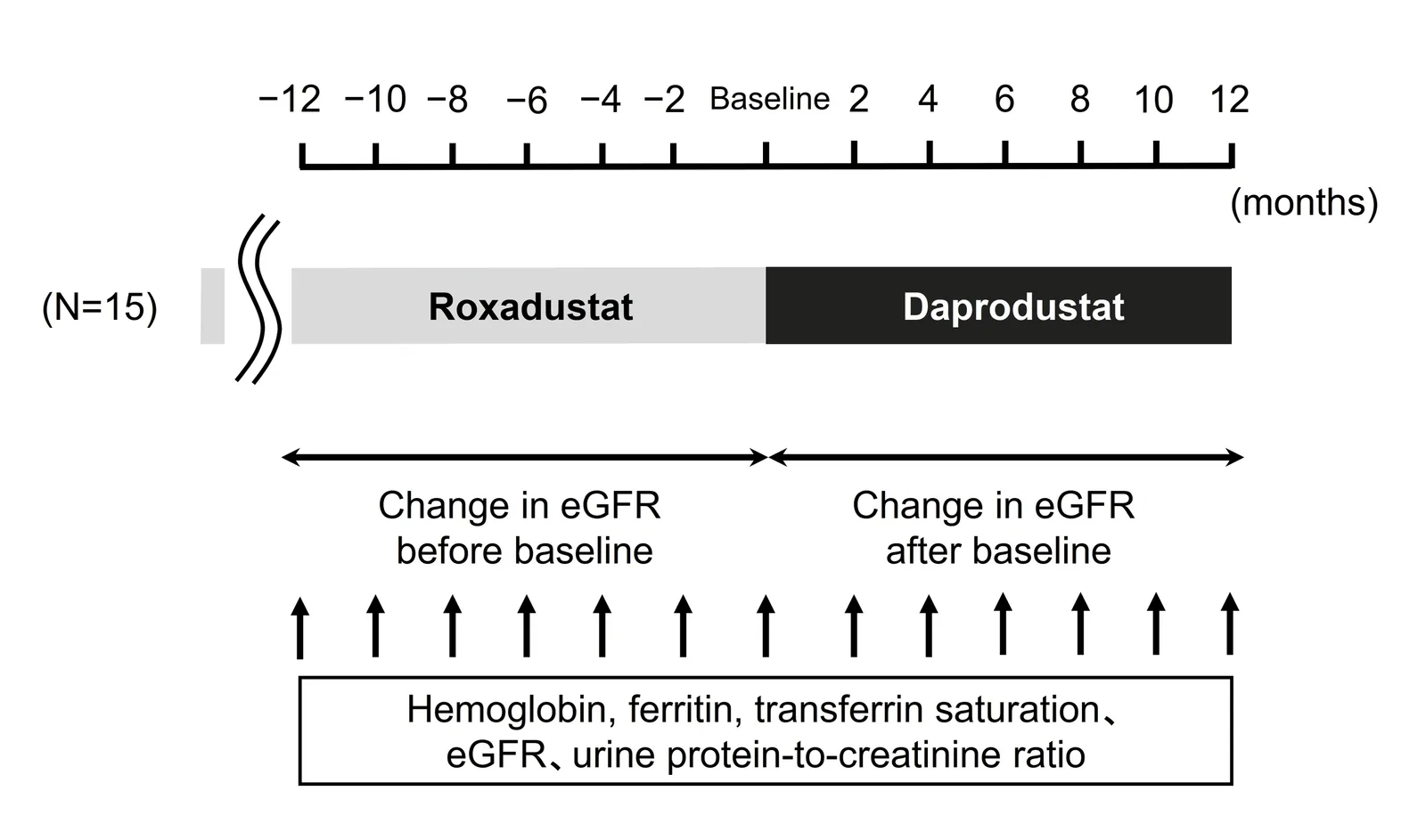
Study design eGFR, estimated glomerular filtration rate. This figure was created using Microsoft PowerPoint (Microsoft Corporation, Redmond, WA, USA).

The study was a retrospective observational study consisting of 15 patients. The study design diagram is illustrated in Figure 1. Roxadustat and daprodustat doses (mg/administration) were recorded according to dosing regimens based on previous clinical trials [8-11]. The dosing regimen of roxadustat in this study followed the methodologies described in phase 3 clinical trials [8,9]. Similarly, the dosing regimen of daprodustat was based on phase 3 clinical trial methodologies [10,11]. Roxadustat was administered orally thrice weekly, at bedtime. Daprodustat was administered orally once daily, after breakfast. Changes in hemoglobin, serum ferritin, transferrin saturation (TSAT), and the doses of roxadustat and daprodustat were evaluated between 12 months before and 12 months after baseline. The dose conversion ratio between roxadustat and daprodustat was calculated using the roxadustat dose at -2 months and the daprodustat dose at 12 months, based on a previous study that established a dose conversion ratio between epoetin alfa and darbepoetin alfa [12]​​​​​​​. The changes in eGFR and urine protein-to-creatinine ratio were also evaluated. The eGFR change rate was evaluated 12 months before and after baseline.

Laboratory methods

Blood and urinary parameters were determined in the hospital clinical laboratory. eGFR was calculated using the equation formula provided by the Japanese Society of Nephrology; eGFR=194 × (serum creatinine) ^−1.094 ^× (age) ^−0.287^ × 0.739 (if female) [13]​​​​​​​. The eGFR change rate was determined as the slope per month for each patient before and after baseline using linear regression analysis [14]​​​​​​​. The classification of CKD stages (G1-G5) was performed based on the Japanese Society of Nephrology CKD guideline [7]​​​​​​​. eGFR and CKD staging were calculated and classified using equations and criteria published by the Japanese Society of Nephrology, which are freely available and widely used in clinical practice [7,13]​​​​​​​.

Statistical analyses

Statistical analysis was performed using JMP 11 (SAS Institute Inc., Cary, NC, USA) under institutional license. Data were expressed as means ± standard deviations for continuous variables, and as counts and percentages for categorical variables. Comparisons of clinical parameters before and after baseline were performed using repeated-measures analysis of variance with Tukey’s test. Comparison of the eGFR change rate before and after baseline was performed using the paired t-test.

Data visualization

All figures and tables were created exclusively using Microsoft Excel and/or Microsoft PowerPoint (Microsoft Corporation, Redmond, WA, USA) under institutional license.

## Results

Patient characteristics

Initially, 26 patients with non-dialysis CKD who were switched from roxadustat to daprodustat were identified. Of these, 11 patients were excluded on the basis of the inclusion and exclusion criteria. Therefore, we analyzed the data of 15 patients (5 men and 10 women, mean age: 76.5 ± 9.3 years, mean body mass index: 23.7 ± 3.9 kg/m^2^). The mean baseline eGFR was 14.3 ± 9.4 mL/min/1.73 m^2^, and their CKD stages were as follows: stage G3a, 0 (0.0%) patient; stage G3b, 1 (6.7%) patient; stage G4, 4 (26.7%) patients; and stage G5, 10 (66.7%) patients. The mean values of hemoglobin concentration, TSAT, and serum ferritin concentration at baseline were 11.2 ± 0.9 g/dL, 26.7 ± 10.3 %, and 86.0 ± 97.8 ng/mL, respectively. The mean roxadustat dose was 51 ± 18 mg/administration. A history of diabetes mellitus was present in 33.3%, hypertension in 93.3%, myocardial infarction in 0.0%, and stroke in 13.3% of the participants. The percentages of participants receiving each medicine were as follows: calcium-containing phosphate binder, 13.3%; calcium-free phosphate binder, 6.7%; vitamin D analog, 20.0%; iron supplement, 20.0%; zinc supplement, 13.3%; and statin, 80.0%. Table 1 summarizes the patient characteristics and medications at baseline.

**Table 1:** Patients’ characteristics and medications Data are presented as means ± standard deviations, or numbers (%). This table was created using Microsoft Excel (Microsoft Corporation, Redmond, WA, USA). Estimated glomerular filtration rate and chronic kidney disease staging were calculated and classified using equations and criteria published by the Japanese Society of Nephrology [11,13].

		All patients (n=15)
Age (years)		76.5 ± 9.3
Male sex (number, %)		5 (33.3%)
Body mass index (kg/m^2^)		23.7 ± 3.9
Diabetes mellitus (number, %)		5 (33.3%)
Hypertension (number, %)		14 (93.3%)
Previous myocardial infarction (number, %)		0 (0.0%)
Previous stroke (number, %)		2 (13.3%)
Calcium-containing phosphate binder (number, %)		2 (13.3%)
Calcium-free phosphate binder (number, %)		1 (6.7%)
Vitamin D analog (number, %)		3 (20.0%)
Iron supplement (number, %)		3 (20.0%)
Zinc supplement (number, %)		2 (13.3%)
Statin (number, %)		12 (80.0%)
Serum creatinine (mg/dL)		4.0 ± 2.2
Estimated glomerular filtration rate (ml/min/1.73m^2^)		14.3 ± 9.4
Chronic kidney disease stage (number, %)	G3a	0 (0.0%)
	G3b	1 (6.7%)
	G4	4 (26.7%)
	G5	10 (66.7%)
Urine protein-to-creatinine ratio (g/gCr)		2.77 ± 2.61
Hemoglobin A1c (%)		5.8 ± 0.7
Total cholesterol (mg/dL)		135.5 ± 21.3
Low-density lipoprotein-cholesterol (mg/dL)		62.0 ± 17.3
High-density lipoprotein-cholesterol (mg/dL)		48.3 ± 11.0
Triglyceride (mg/dL)		96.2 ± 45.5
Uric acid (mg/dL)		5.5 ± 1.4
Albumin (g/dL)		3.5 ± 0.3
Hemoglobin (g/dL)		11.2 ± 0.9
Sodium (mEq/L)		139.1 ± 2.7
Potassium (mEq/L)		4.9 ± 0.7
Chloride (mEq/L)		106.9 ± 3.0
Total calcium (mg/dL)		8.9 ± 0.5
Phosphorus (mg/dL)		4.4 ± 0.8
Magnesium (mg/dL)		2.0 ± 0.4
C-reactive protein (mg/dL)		0.83 ± 1.62
Transferrin saturation (%)		26.7 ± 10.3
Ferritin (ng/mL)		86.0 ± 97.8
Roxadustat dose (mg/administration)		51 ± 18

Change in hemoglobin concentration

**Figure 2:**
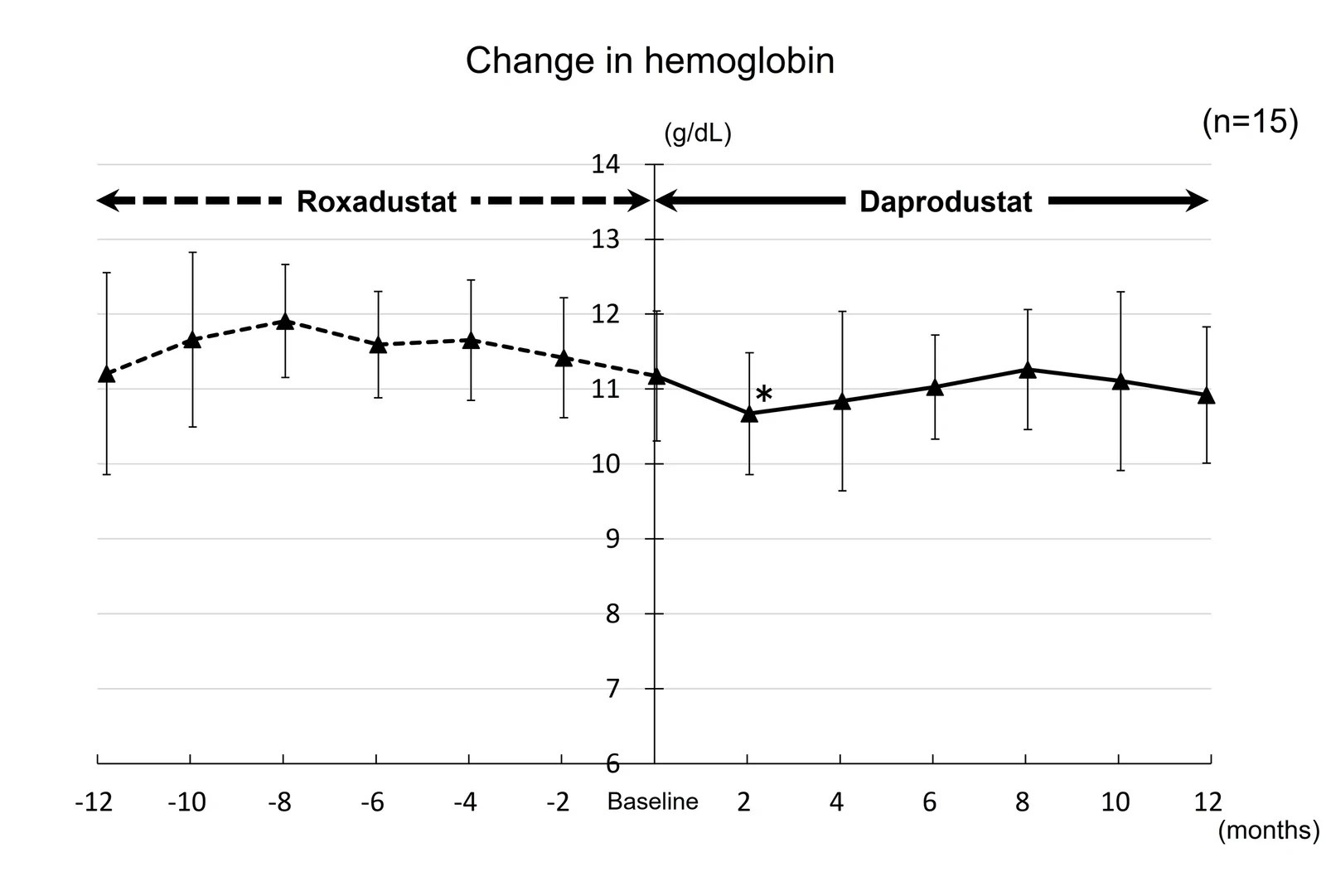
Change in the hemoglobin levels during the study *, p < 0.05 vs. -8 months. This figure was created using Microsoft Excel and Microsoft PowerPoint (Microsoft Corporation, Redmond, WA, USA).

Hemoglobin concentration was significantly lower at two months than at -8 months (p < 0.05). Hemoglobin concentration did not significantly change after baseline (Figure 2).

Change in iron metabolism

**Figure 3:**
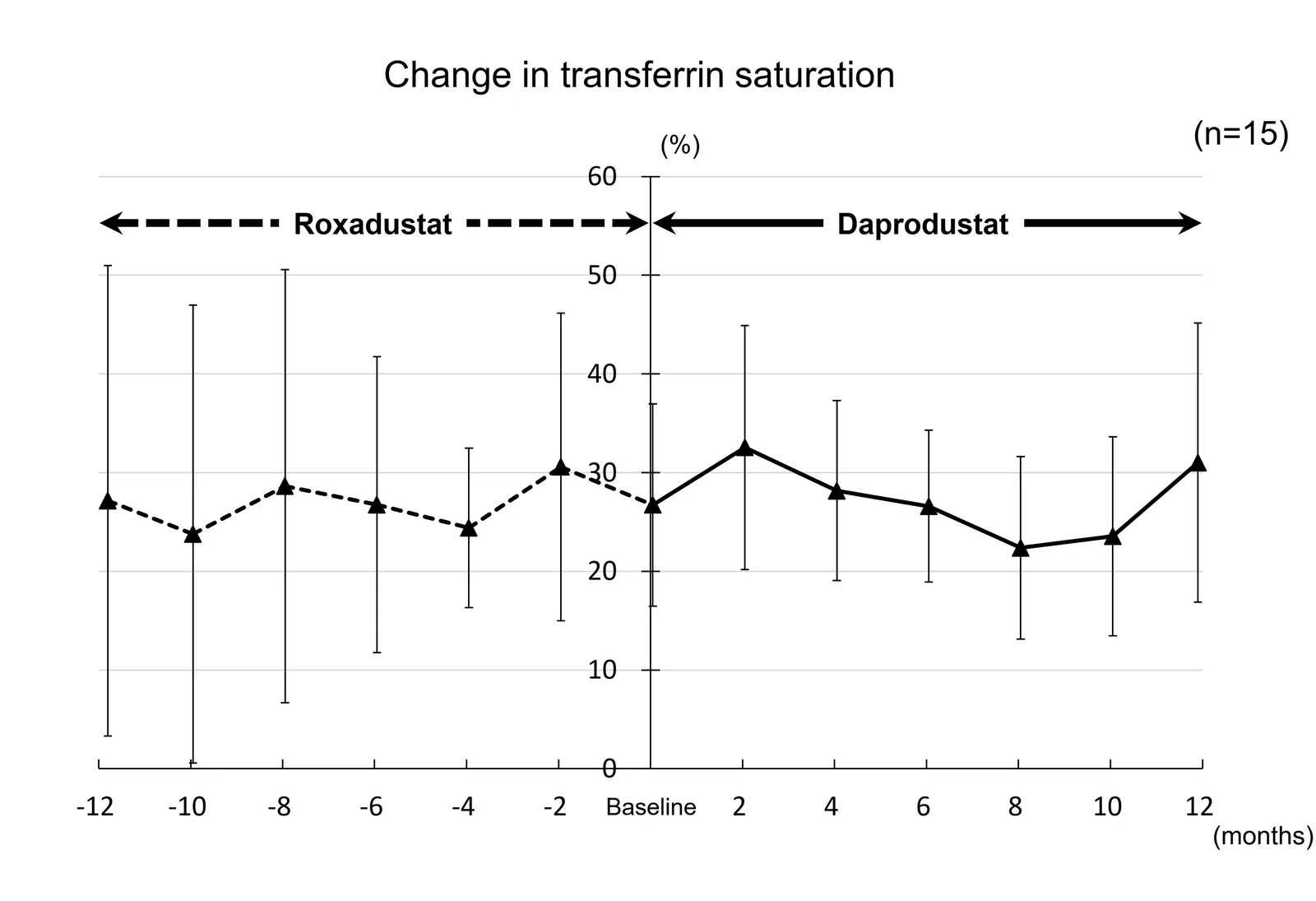
Change in the transferrin saturation during the study This figure was created using Microsoft Excel and Microsoft PowerPoint (Microsoft Corporation, Redmond, WA, USA).

**Figure 4:**
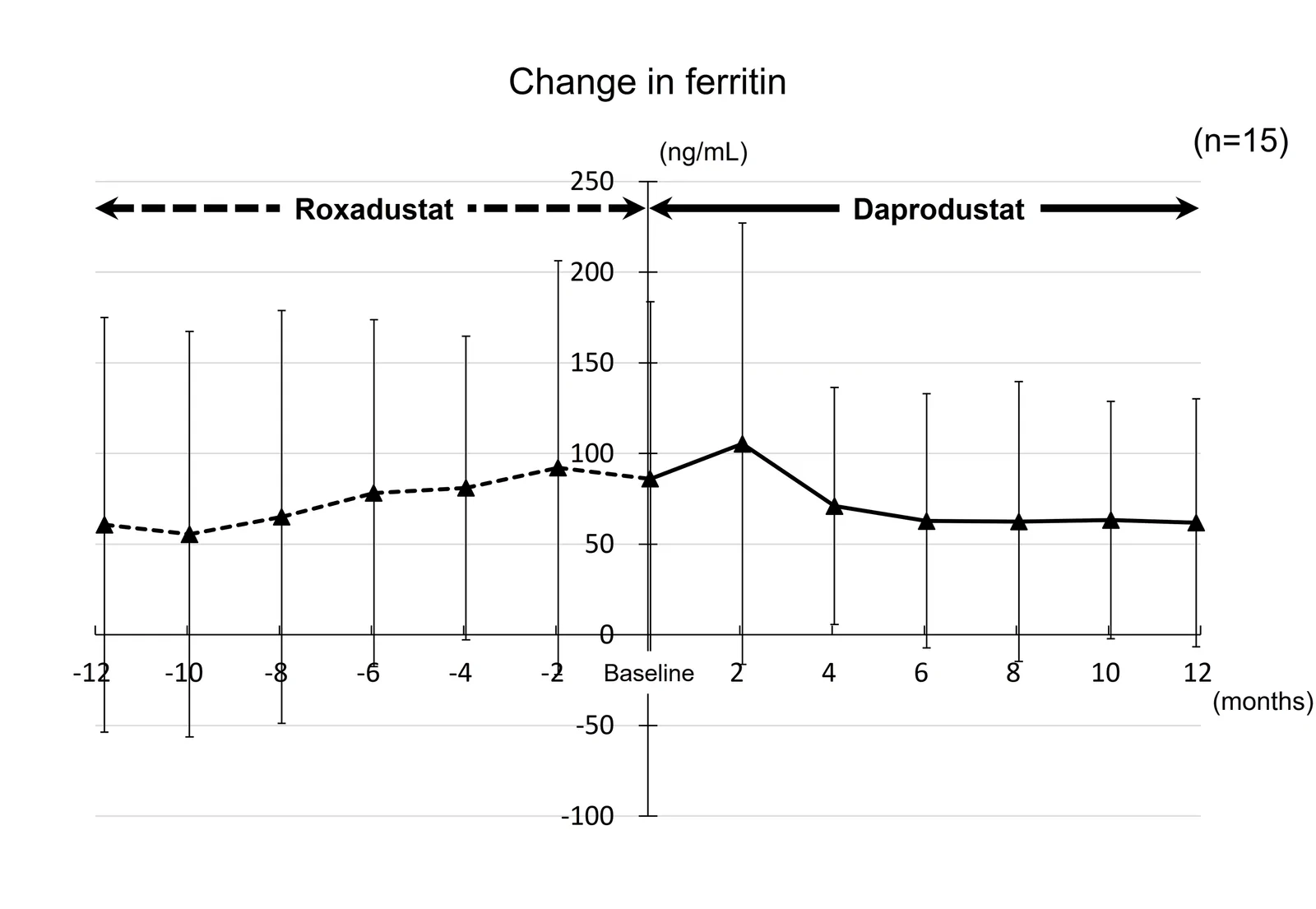
Change in the serum ferritin levels during the study This figure was created using Microsoft Excel and Microsoft PowerPoint (Microsoft Corporation, Redmond, WA, USA).

TSAT did not significantly change during the study period (Figure 3). Similarly, serum ferritin concentration showed no significant change during the study period (Figure 4).

Changes in roxadustat and daprodustat doses

**Figure 5:**
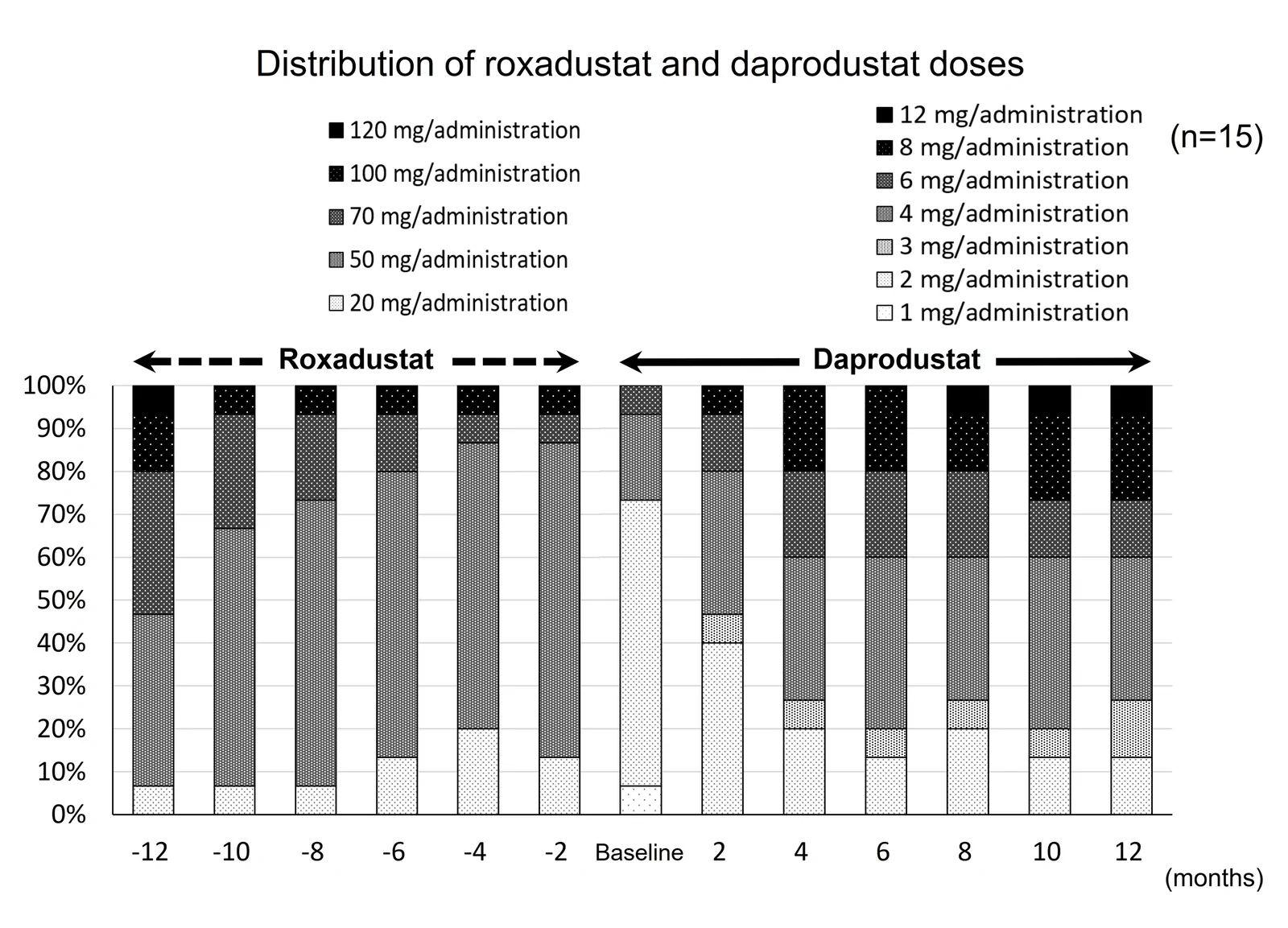
Distribution of roxadustat and daprodustat doses administered during the study This figure was created using Microsoft Excel and Microsoft PowerPoint (Microsoft Corporation, Redmond, WA, USA).

**Figure 6:**
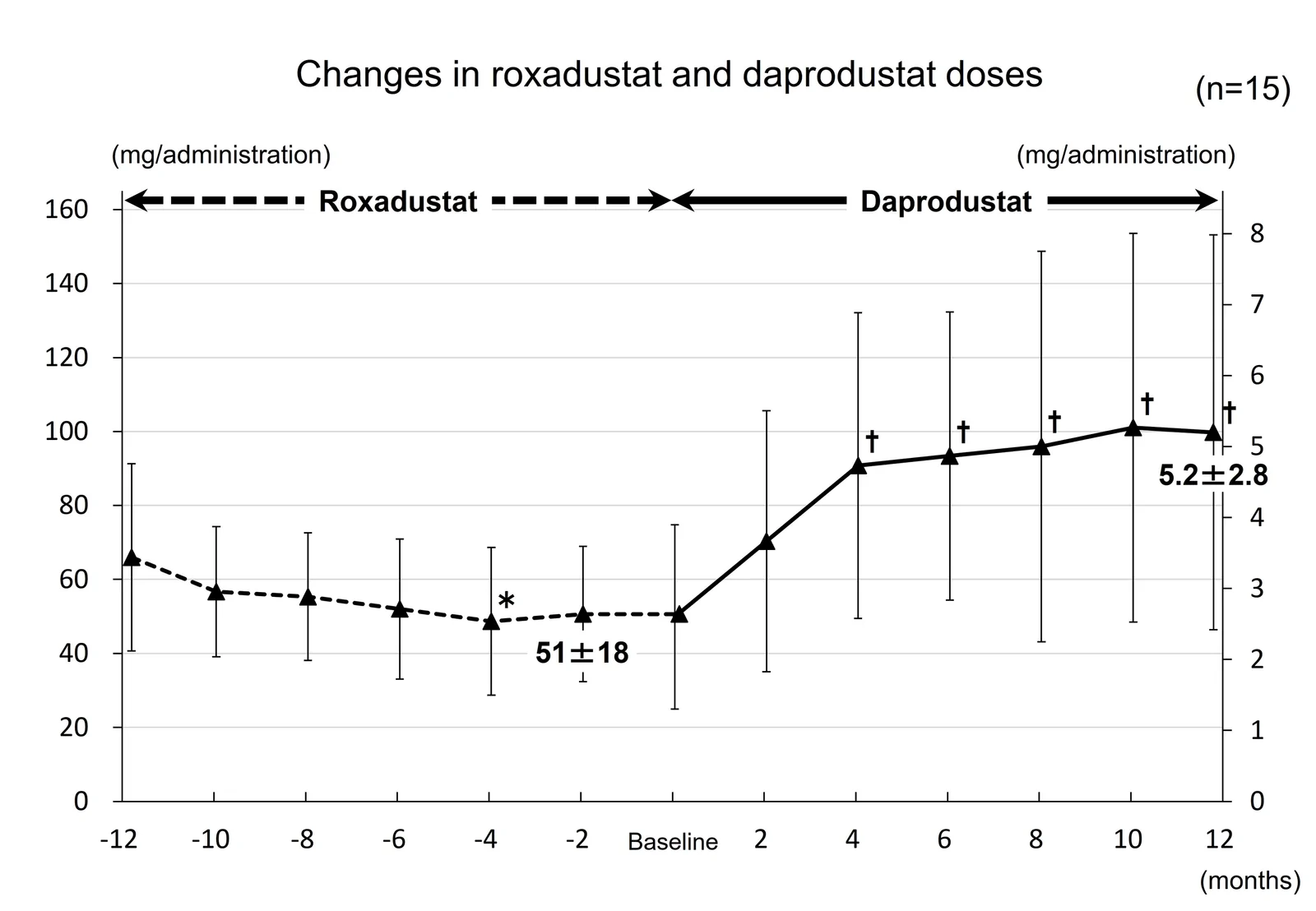
Changes in the roxadustat and daprodustat doses administered during the study *, p < 0.05 vs. -12 months; †, p < 0.05 vs. baseline. This figure was created using Microsoft Excel and Microsoft PowerPoint (Microsoft Corporation, Redmond, WA, USA).

The distributions of roxadustat and daprodustat doses are presented in Figure 5. The changes in doses of roxadustat and daprodustat are shown in Figure 6. The changes in doses of roxadustat and daprodustat in each patient are shown in Table 2. The dose of roxadustat decreased significantly from 66 ± 25 mg/administration at -12 months to 49 ± 20 mg/administration at -4 months (p < 0.05). The dose of daprodustat increased significantly from 2.6 ± 1.3 mg/administration at baseline to 4.7 ± 2.2 mg/administration at four months (p < 0.05), 4.9 ± 2.0 mg/administration at six months (p < 0.05), 5.0 ± 2.8 mg/administration at eight months (p < 0.05), 5.3 ± 2.7 mg/administration at 10 months (p < 0.05), and 5.2 ± 2.8 mg/administration at 12 months (p < 0.05). Therefore, the dose conversion ratio between roxadustat and daprodustat was calculated as follows: roxadustat dose at -2 months (51 mg/administration) ÷ daprodustat dose at 12 months (5.2 mg/administration) ≈ 10:1.

**Table 2:** The changes in doses of roxadustat and daprodustat in each patient M, month. Rows were sorted based on the roxadustat dose at -2 months and the daprodustat dose at 12 months. This table was created using Microsoft Excel (Microsoft Corporation, Redmond, WA, USA).

Patient number	Roxadustat dose (mg/administration)						Daprodustat dose (mg/administration)						
	-12M	-10M	-8M	-6M	-4M	-2M	Baseline	2M	4M	6M	8M	10M	12M
1	50	50	50	50	20	20	2	4	4	4	4	4	4
2	20	20	20	20	20	20	2	4	8	8	8	8	8
3	50	50	50	20	20	50	2	2	2	2	2	2	2
4	70	70	70	50	50	50	2	3	3	3	2	2	2
5	70	70	50	50	50	50	2	2	4	4	3	3	3
6	50	50	50	50	50	50	2	4	4	4	4	4	4
7	50	50	50	50	50	50	2	4	4	4	4	4	4
8	70	70	70	50	50	50	1	2	2	2	2	4	4
9	100	50	50	50	50	50	2	2	6	6	6	4	4
10	70	70	70	70	50	50	4	6	6	6	6	6	6
11	70	50	50	50	50	50	2	2	4	4	4	8	8
12	100	50	50	50	50	50	4	4	8	8	8	8	8
13	50	50	50	50	50	50	4	8	8	8	12	12	12
14	50	50	50	70	70	70	2	2	2	4	4	4	3
15	120	100	100	100	100	100	6	6	6	6	6	6	6

Changes in eGFR and urine protein-to-creatinine ratio

**Figure 7:**
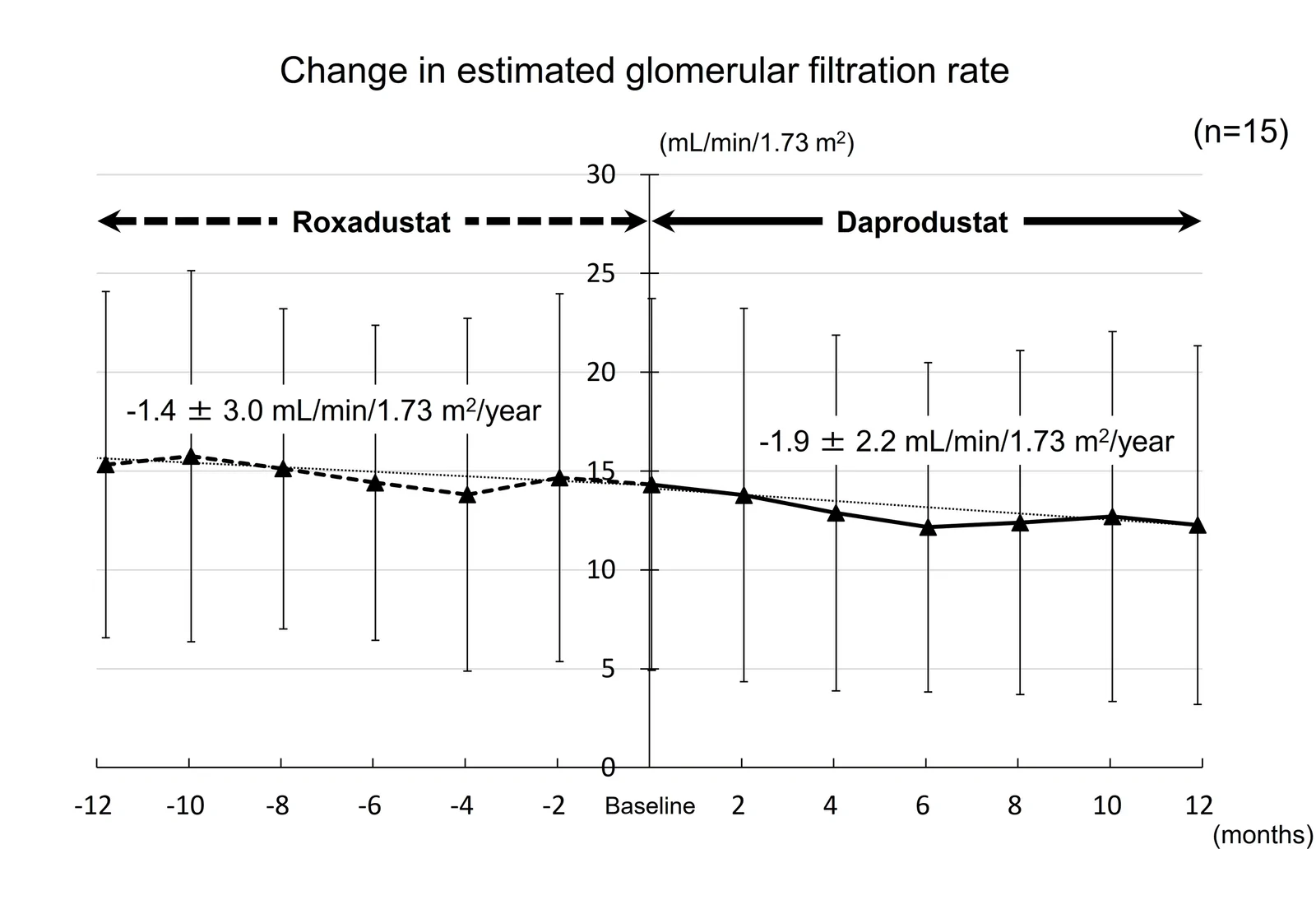
Change in the estimated glomerular filtration rate during the study This figure was created using Microsoft Excel and Microsoft PowerPoint (Microsoft Corporation, Redmond, WA, USA).

**Figure 8:**
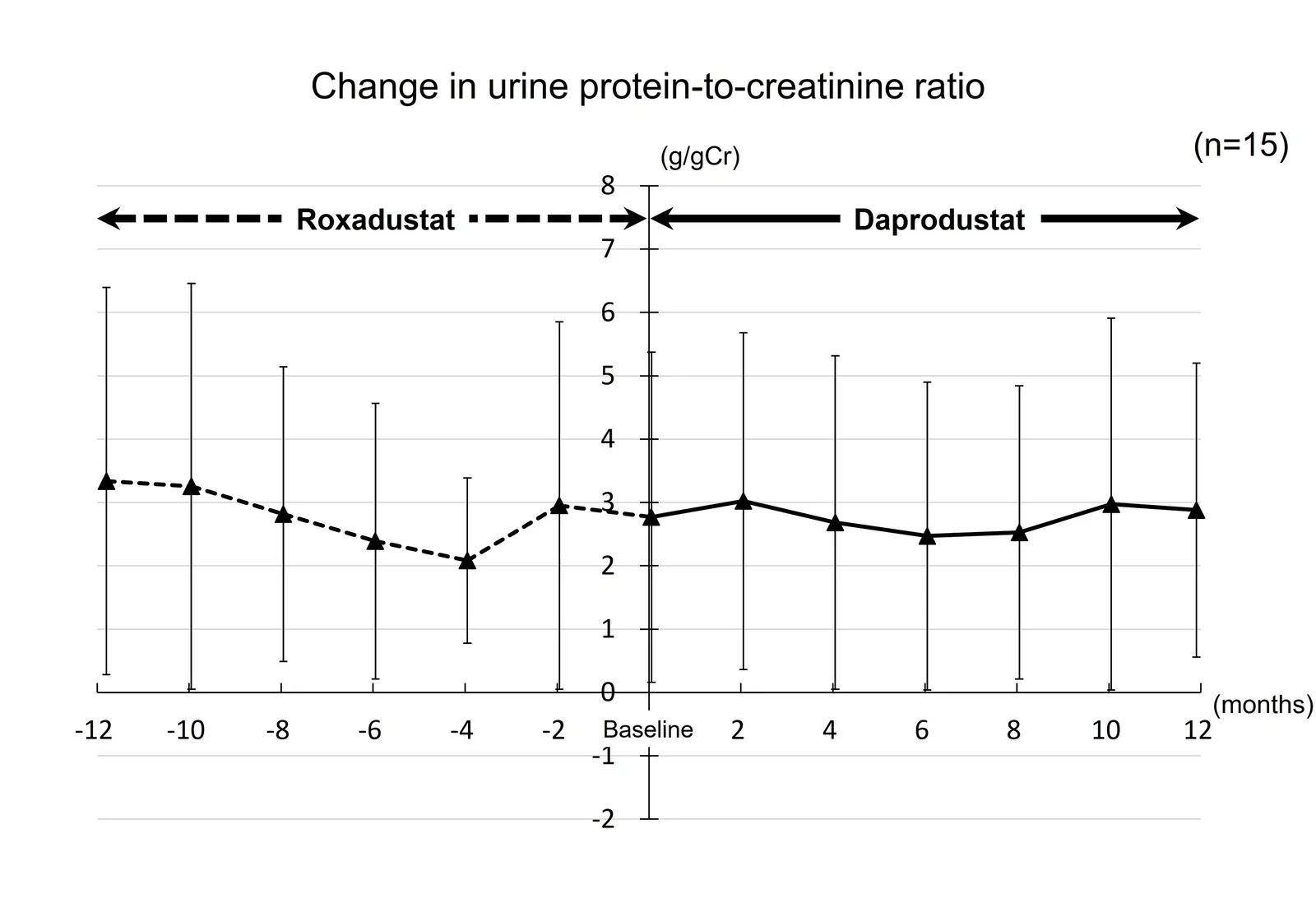
Change in the urinary protein-to-creatinine ratio during the study This figure was created using Microsoft Excel and Microsoft PowerPoint (Microsoft Corporation, Redmond, WA, USA).

The eGFR change rate did not significantly differ between before and after baseline (Figure 7). The urine protein-to-creatinine ratio did not significantly change over the study period (Figure 8).

Changes in other clinical parameters

Other laboratory parameters, including hemoglobin A1c, total cholesterol, low-density lipoprotein cholesterol, high-density lipoprotein cholesterol, triglyceride, uric acid, albumin, sodium, potassium, chloride, total calcium, phosphate, magnesium, and C-reactive protein were not signiﬁcantly different during the study period.

## Discussion

In the present study, we determined the dose conversion ratio between roxadustat and daprodustat in non-dialysis CKD patients. The identified conversion ratio of approximately 10:1 may assist clinicians when switching from roxadustat to daprodustat in CKD patients.

Previous randomized controlled trials have demonstrated robust hemoglobin-raising effects of roxadustat and daprodustat in non-dialysis CKD patients [8-11]. A network meta-analysis reported that roxadustat was superior to daprodustat with respect to its effect on anemia in CKD patients [5]. In the present study, the hemoglobin level decreased after switching from roxadustat to daprodustat, which necessitated dose escalation of daprodustat. These findings suggest that the dose escalation of daprodustat is necessary when roxadustat is switched to daprodustat in CKD patients. Differences in effective dosing between roxadustat and daprodustat may be explained by variations in pharmacokinetics and HIF stabilization profiles, despite their shared mechanism of action as HIF-PH inhibitors [15]. The aforementioned network meta-analysis showed that the effect on iron metabolism did not differ between roxadustat and daprodustat in CKD patients [5]​​​​​​​. In the present study, both TSAT and serum ferritin concentration remained almost unchanged after switching from roxadustat to daprodustat. These findings suggest that the dose adjustment of iron supplements is not necessary when roxadustat is switched to daprodustat in CKD patients.

There are few studies that have examined the dose conversion ratio between HIF-PH inhibitors. An observational study compared the changes in hemoglobin levels and doses between vadadustat and daprodustat in non-dialysis CKD patients [16]​​​​​​​. There was no difference in hemoglobin levels between the vadadustat group and daprodustat group during the 12 months of study period. The dose of daprodustat did not change during the study period (4 mg/day), whereas the dose of vadadustat increased by 70% during the study period (300 mg/day to 510 mg/day). These findings indicate a relative potency ratio favoring daprodustat over vadadustat. To the best of our knowledge, this is the first study to determine the dose conversion ratio between roxadustat and daprodustat. This conversion ratio can be practically applied when switching from roxadustat to daprodustat or from daprodustat to roxadustat in non-dialysis CKD patients, allowing clinicians to estimate an appropriate starting dose of the alternative HIF-PH inhibitor and thereby facilitate safer and more efficient dose adjustment. Our study may provide the foundation for additional research to investigate the dose conversion ratio between roxadustat and daprodustat.

This study had several strengths. First, it addressed a practical and clinically relevant issue, the dose conversion between roxadustat and daprodustat, which is directly applicable to anemia management in non-dialysis CKD patients. Second, the use of real-world clinical data and a longitudinal observational design enhances the clinical relevance of the findings in an area where existing evidence remains limited. Third, the focused and pragmatic analytical approach enables interpretation within routine clinical practice.

This study had several limitations. First, the retrospective observational study design may have introduced patient selection bias. Second, the very low participant number (n=15) and single-center study design limit generalizability. Third, the before-after design lacked a control group. Fourth, only non-dialysis CKD patients were included, and the results may not be applicable to patients receiving dialysis. Given the retrospective and observational design of this study and only fifteen participants recruited from a single institution in one country, the study results should be interpreted with caution. Therefore, large-scale prospective studies with an appropriate control group are required to confirm the dose conversion ratio between roxadustat and daprodustat in CKD patients.

## Conclusions

Our study results suggest that the dose conversion ratio between roxadustat and daprodustat is approximately 10:1 in non-dialysis CKD patients. This conversion ratio may provide a foundation for future prospective studies and assist clinicians when switching between these agents. Further large-scale prospective studies are warranted to validate this conversion ratio and to clarify its generalizability.
